# High Spectral Sensitivity of Strongly Coupled Hybrid Tamm-Plasmonic Resonances for Biosensing Application

**DOI:** 10.3390/s22239453

**Published:** 2022-12-03

**Authors:** Justina Anulytė, Ernesta Bužavaitė-Vertelienė, Evaldas Stankevičius, Kernius Vilkevičius, Zigmas Balevičius

**Affiliations:** Plasmonics and Nanophotonics Laboratory, Department of Laser technology, Center for Physical Sciences and Technology, Sauletekio Ave. 3, LT-10257 Vilnius, Lithuania

**Keywords:** hybrid lattice plasmonic resonance, strong coupling, Tamm plasmons

## Abstract

In this study, the sensitivity to the refractive index changes of the ambient was studied on the uniform gold film (~50 nm) with a 1D photonic crystal (PC) from periodic five TiO_2_ (~110 nm)/SiO_2_ (~200 nm) bilayers and gold nano-bumps array produced by direct laser writing on the same sample. The optical signal sensitivity of hybrid plasmonic resonances was compared with traditional surface plasmon resonance (SPR) on a single gold layer. The influence of the strong coupling regime between Tamm plasmon polariton (TPP) and propagated plasmon polaritons in the hybrid plasmonic modes on the sensitivity of the optical was discussed. Recent studies have shown very high hybrid plasmonic mode sensitivity S_HSPP_ ≈ 26,000 nm/RIU to the refractive index on the uniform gold layer; meanwhile, the introduction of gold lattice reduces the signal sensitivity, but increases the Q-factor of the plasmonic resonances. Despite this, the sensitivity to the ellipsometric parameters Ψ and Δ on the gold lattice was rather high due to the increased Q-factor of the resonances. The comparison of plasmonic resonance sensitivity to the refractive index changes of hybrid TPP-SPP mode on the uniform gold layer and traditional SPR have shown that hybrid plasmonic mode, due to a strong coupling effect, overcomes the SPR by about 27%.

## 1. Introduction

Light interaction with metals at the nanoscale demonstrates enhancement of local electromagnetic field at the metal-dielectric interfaces [1] and is generally known as plasmonics. Plasmons are collective oscillations of conduction electrons in the metal and can couple with light photons, forming the polariton state. The generation of plasmon polaritons leads to high localization and enhancement of the electric field of light at the optical frequencies. More than three decades ago, surface plasmon resonance was successfully applied for optical biosensors studying protein interaction [2]. Later various structures based on plasmonic effects were studied for different possible applications, such as label-free single molecule and optical sensing [3,4], perfect absorbers [5], fast switching [6], as well as novel plasmonic lasing [7] in room temperature. These applications of various plasmonic resonances under certain conditions could be achieved in structures such as metallic nanoparticles [8], thin metal layers [9], or metallic nanostructures [10]. One of the excitations that has been widely used for biosensing applications is the propagating surface plasmon resonance, generated on the thin metal layer (up to 50 nm). The SPR has greater wave-vector than a photon in the free space for the same frequency due to the wave-vector matching optical elements, such as prism or grating [9]. The Kretschmann (prism) configuration is one of the most popular, p-polarized light incidents to a glass prism, which is then reflected from the thin metal layer deposited on the prism base. The surface plasmon polariton wave is excited at the outer side of the metal film for a specific wavelength and angle of incidence (AOI). The SPR resonance manifested themselves as a dip in the reflection spectra. The localized surface plasmons (LSP) are the oscillations of free electrons on the metallic nanoparticles. The resonance frequency of LSP depends on size, shape, and local dielectric function of the media. Such plasmonic resonances mostly occur from optical to near-infrared region and do not require wave vector matching couplers (prism, grating, or waveguide) or periodic photonic structures (Bragg reflector). However, LSPR usually has a wider width of the resonances than SPR, which indicates lower losses of the latter.

Another plasmonic excitation generated in a 1D photonic crystal structure with a thin metal layer on top is the Tamm plasmons polariton (TPP), which were applied for narrow band tunable filters [11] and biosensors [12,13]. The Tamm plasmon polaritons are non-propagating optical states that exist at the metal and photonic crystal boundary. The confined optical state of TPP in the metal is formed due to the negative dielectric permittivity of the metal that is the same as the SPP; meanwhile, the electric field confinement in the periodic structure of the Bragg mirror is achieved because of the photonic stop band of the 1D photonic crystal. One of the main features of TPP is its in-plane wave-vector, which is smaller than the wave vector of light in a vacuum; thus, the TPPs can be directly excited with incident light without a prism coupler, contrary to the SPPs, which have a wave vector larger than the one of the incident light [14]. However, the plasmonic excitations, such as SPPs or randomly distributed nanoparticles have high energy losses due to metal. In order to minimize the energy losses or in cases when metal surfaces cannot be used, the periodic dielectric structures of Bragg reflectors can be used in total internal reflection (TIR) configuration for excitation of Bloch surface waves (BSW). The optical dispersion of these surface waves are below the light cone which is similar to the SPP. Thus, BSW share some of the optical features [15] as the SPP, and therefore they are used widely for optical biosensing in TIR configuration as SPR biosensors [16,17]. It has been shown that spectral sensitivity of the BSW biosensors is lower than SPR; however, due to lower losses in periodic dielectric structures the angular sensitivity of reflected polarized intensity or ellipsometric parameters was better than for SPR [16]. Moreover, the energy density of the BSW in dielectric periodic structures were theoretically modelled by applying the zero admittance approach [18] and further changes in the spatial profile of the reflected laser beam intensity were experimentally measured [19]. The sensitivity of Δn ≈ 10^−5^ for refractive index measurements was achieved, which is typical for commercially available SPR devices. Recently, it has been shown that using various metallic nanostructures (grating arrays) arranged in a periodic manner allows us to minimize losses in the metals. As a result, such metallic arrays compared with randomly distributed nanoparticles or SPPs exhibit narrow plasmonic resonances [20] with an increased quality factor Q. It was shown that the surface lattice arrays influence the propagation length of the hybrid Tamm surface plasmon polaritons modes under strong coupling [21]. The decreasing losses were also achieved in hybrid Tamm plasmon polariton modes due to strong coupling between them [22]. By achieving the conditions needed for both plasmonic excitations, a new state of hybrid TPP-SPP mode appears [23]. These hybrid TPP-SPP modes can be realized in structures consisting of PC with a thin metal layer on the top by optically connecting a glass prism to the PC. The light-matter interaction in such structures can lead to strong coupling between the TPP and SPP components in the hybrid mode and the components become inextricably linked with each other [24,25]. If the strong coupling regime is achieved, the energy exchange between the TPP and SPP modes occur during a coherent time that is about tens of fs.

As mentioned above, narrow plasmonic resonances with lower energy losses have been demonstrated on periodically arranged metallic nanostructure arrays [26]. The current lithography and direct laser writing [27] methods enable the production of complex nanostructures supporting multiple plasmonic modes, which can be analyzed as the interaction of elementary plasmons supported by the nanostructures. The most popular nanostructures of complex plasmonic systems used are the arrays of metallic nanoparticles with a period that is by size comparable with the wavelength of the incident light [28]. Such nanostructures exhibit a hybridization effect of the plasmonic resonances where different localized surface plasmons (LSPs) of the nanoparticles interact with each other [29]. The damping of the localized plasmon resonance is compensated by the scattered field of light [10] in the individual particles; as a result this led to a significant narrowing of the plasmonic resonance. For surface plasmon resonance biosensors the Kretschmann configuration with glass prism was applied for most cases [3,30,31]. The attempts to increase the sensitivity of the SPR sensors led to employing the phase measurements by using ellipsometry [32], where the amplitude (Ψ) and phase (Δ) of ellipsometric parameters for light reflected from the sample can be obtained. The ratio of reflected polarized amplitudes p- and s- polarization gives ellipsometric parameter Ψ, meanwhile the difference between them describes phase shift Δ. The combination of an ellipsometric optical scheme with a glass prism gives ellipsometric measurements under a total internal reflection [33]. Such total internal reflection ellipsometry (TIRE) gives more sensitive phase measurements, and ellipsometric parameter Ψ for SPR resonance has a narrower FWHM width compared with conventional intensity measurements, which also give better sensitivity characteristics. It was shown that an abrupt phase jump occurs at the plasmonic resonances when reflection intensity drops to zero (or so-called topological darkness); however, it is difficult to achieve it experimentally due to the surface roughness and other non-idealities of the sample [3]. TIRE method was also widely tested for the study of protein interactions [34,35]. 

The optical dispersion features of 1D PC with a uniform gold layer and nano-bumps array were analyzed and compared by using total internal reflection ellipsometry (TIRE). In this study, the sensitivity to the refractive index changes of the ambient was studied on the uniform gold film with 1D PC with periodic five TiO_2_/SiO_2_ bilayers and gold nano-bumps array produced by direct laser writing on the same sample. The optical signal sensitivity of hybrid plasmonic resonances was compared with traditional surface plasmon resonance (SPR) on a single gold layer. The influence of the strong coupling regime between Tamm and propagated plasmon polaritons in the hybrid plasmonic modes on the sensitivity of the optical sensors was discussed. In addition, the contribution of gold nano-bumps to the Q-factor of plasmonic resonances was estimated.

## 2. Materials and Methods

The Au film was deposited on PC using the magnetron sputtering method and then the gold micro bumps were produced by using second-harmonic (515 nm) of 300-fs laser pulses generated by Yb:KGW based fs-laser (Pharos, Light Conversion Ltd. Vilnius, Lithuania). The femtosecond laser beam was tightly focused in ~1 µm spot with an objective having the numerical aperture (NA) of 0.5. The sample translation speed and pulse repetition rate were selected 2.8 mm s^−1^ and 4 kHz, respectively, to keep a 0.7 μm distance between bumps in the scanning direction (*x*-axis). The distance between bumps in the *y*-axis direction (perpendicular to beam scanning) was selected 0.7 μm by moving the translation stage. Each gold bump was fabricated using a single laser pulse with 0.5 nJ energy. The nano-bumps were formed using a lower pulse energy than in our previous work [27], as they were formed on a photonic crystal rather than a glass substrate. The early works of the metallic films modification by laser pulses studied the influence of various substrates for the formed microstructure’s quality [36]. The quality of micro-bumps produced by laser pulses on the nickel film deposited by evaporation and sputtered technique were compared and the electrical properties were analysed [37]. 

**Figure 1:**
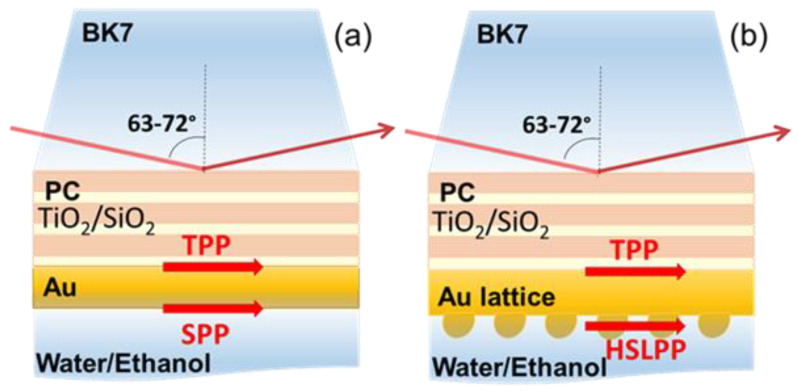
Scheme of excitation configurations of the PC(TiO_2_/SiO_2_)/Au structures for generating the TPP-SPP (**a**) and TPP-HSLPP (**b**) modes by spectroscopic ellipsometry in total internal reflection (TIR).

The penetration depth of the TPP and SPP at the optical wavelengths is about 25–30 nm; thus, the thickness of the metal had to be thin enough (≈40–50 nm) for the TPP and the SLPP components to couple in a hybrid mode. The sample used for the hybrid surface lattice propagated plasmon (HSLPP) mode excitation consisted of a 1D PC and a thin metal layer of gold bumps (~50 nm) produced by direct laser writing (DLW). The PC used was made of 5 alternating TiO_2_ (~110 nm) and SiO_2_ (~200 nm) bilayers deposited onto a BK-7 glass substrate by means of ion beam sputtering (Figure 1). The thickness of the TiO_2_ and SiO_2_ layers is chosen in such a way that a forbidden photonic band (FPB) would be formed in the visible range where the SPP component of the hybrid mode is generated. The total thickness of the Bragg mirror (1D photonic crystal) determines the position of the FPB in the spectra, so that thicker layers move FPB to the IR region. The differences of the refractive indexes of the materials influence the width of the FPB. The number of layers of alternating materials with varying refractive index influence the quality of the photonic stop band. The wavelengths, which are close to four times the optical thickness of the bilayers, and the multiple reflections from these bilayers gives constructive interference. Therefore, multilayer structure works as a highly reflective mirror for the range of wavelengths which lies in the forbidden band.

The measurements of the structure described above were performed using spectroscopic ellipsometry (SE). The ellipsometer used for obtaining the optical response of the structures was a J. A. Woollam RC-2 model with two rotating compensators. The light source of the RC-2 ellipsometer was a Xe lamp with a spectral range of 210–1700 nm. The total internal reflection (TIR) configuration with a 70° prism coupler for the excitation of the hybrid TPP-SPP and TPP-SLPR modes was used. 

**Figure 2:**
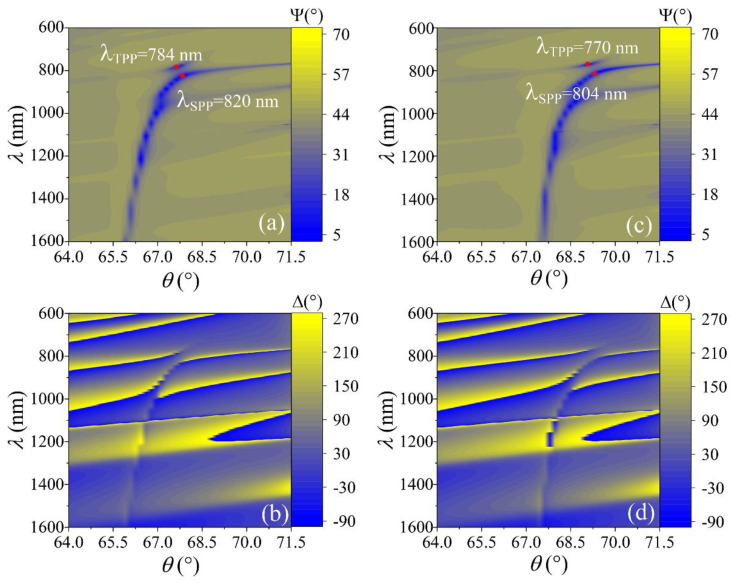
The dispersion maps λ (θ) of ellipsometric parameters Ψ(°) (**a**,**c**) and Δ(°) (**b**,**d**) for structure PC (TiO_2_/SiO_2_ (110 nm/200 nm)/Au (50 nm) in water (left side) and in ethanol (right side). The red points show the strong coupling effect between the Tamm plasmons and the surface plasmon at zero detuning.

The experiment was conducted in two different areas of the same sample: PC with gold film without laser modification and PC with thin gold film modified by direct laser writing. Both the spectra of the PC structure with the gold film without laser modification and the PC structure with gold micro bumps produced by the DLW technique were measured by TIRE. In order to investigate the optical response of the plasmonic resonances due to refractive index changes, a liquid handling system with a custom-built Teflon chamber was used. First, the chamber was filled with deionized water and the TIRE spectra of the ellipsometric parameters Ψ(λ) and Δ(λ) were measured, and then the liquid ambient was changed to ethanol, whose refractive index is higher than that of the pure deionized water. The measured experimental data were expressed as the map of the ellipsometric parameters Ψ(λ, θ) and Δ(λ, θ) dependence on the wavelength (λ) and angle of incidence (θ) (Figure 2). These maps correspond to the dispersion relation of the studied hybrid plasmonic excitations. Furthermore, in order to demonstrate the reduced Ohmic losses of the hybrid TPP-SLPP in TIRE spectra a fixed AOI was chosen.

## 3. Results

The TIRE method was used for the analysis of the optical properties and sensitivity features of the TPP-SPP hybrid modes. As was noted above, two different areas of a sample were investigated: one with a uniform gold layer and another with a nano-bumps array produced by DLW. The TIRE spectra of ellipsometric parameters Ψ and Δ were measured on the sample with non-modified thin metal film and maps presented as wavelength dependence on the AOI (θ = 64–72°). Figure 2 shows the hybrid TPP-SPP mode excitation generated on 1D PC and uniform thin gold film on the top when ambient was deionized water and ethanol. As can be seen from the dispersion maps λ (θ) of ellipsometric parameters Ψ and Δ, the strong coupling effect between the Tamm plasmons and the surface plasmon at the zero detuning point excited at 784 nm and 820 nm in water, and 770 nm and 804 nm in ethanol, respectively. For the hybrid TPP-SPP modes, the SPP component of ellipsometric parameter Ψ become narrower due to the anti-crossing which lies in the λ = 780–800 nm spectral range for the angle of incidence θ = 67–69°. It has been shown that this narrowing of resonance is related to decreased losses of the hybrid plasmonic modes and strong coupling between Tamm and the propagated surface plasmon polaritons, described reasonably well by the simple two coupled oscillators model [22]. The maps of the ellipsometric parameter Δ show sharper dispersion lines of the resonances (Figure 2b,d). The hybrid plasmonic excitation of the TPP and SPP at a fixed angle of incidence (θ = 67.6°) was λ_TPP_ = 783 nm and λ_SPP_ = 843 nm for the deionized water and was λ_TPP_ = 801 nm and λ_SPP_ = 1401 nm for the ethanol at the same AOI. The shifts were δλ_TPP_ = 18.2 nm and δλ_SPP_ = 559.7 nm in the different ambient. The spectral shift of TPP and SPP resonances was caused by the refractive index change of the ambient δn_(λ=783nm)_ = 1.3495 − 1.3286 = 0.0209 and δn_(λ=843nm)_ = 1.3486 − 1.3276 = 0.021, respectively. The refractive index values for deionized water and ethanol were taken for CompleteEase ellipsometric software database [38]. Spectral shift of these resonances gave the corresponding sensitivities to the refractive index unit (RIU) δλ_TPP_/δn = 18.2/0.0209 ≈ 871 nm/RIU and δλ_SPP_/δn = 559.7/0.021≈26 600 nm/RIU. It should be noted that the sensitivity of the conventional SPR sensor with a single thin (~50 nm) gold layer on the glass prism base to refractive index of the ambient was about 19,000 nm/RIU when the ambient was changed from deionized water to ethanol (the results not shown). This number is higher than earlier reported for wavelength investigation (13 800 nm/RIU) [9].

**Figure 3:**
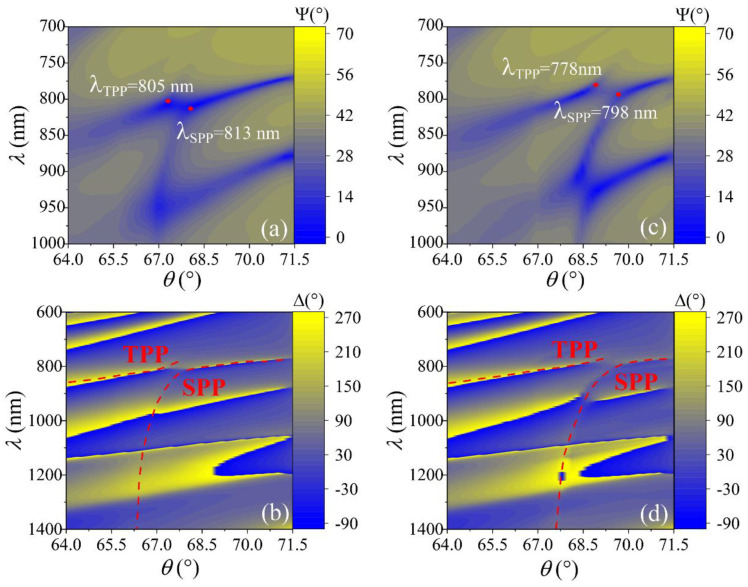
The dispersion maps λ (θ) of ellipsometric parameters Ψ(°) (**a**,**c**) and Δ(°) (**b**,**d**) for structure PC (TiO_2_/SiO_2_ (110 nm/200 nm)/with gold nano-bumps (50 nm) in water (left side) and in ethanol (right side). The red points show the strong coupling effect between the Tamm plasmons and the surface plasmon at zero detuning.

The TIRE spectra of ellipsometric parameters Ψ and Δ (θ = 64–72°) were also measured in the area with nano-bumps grating formed by the DLW method. As can be seen from Figure 3, the ellipsometric parameters Ψ and Δ optical dispersion maps λ(θ), the anti-crossing effect remains at the same energies as the uniform layer; however, an additional dispersion line appears between the TPP and SPP components (λ_TPP_ = 805 nm and λ_SPP_ = 813 nm in water, λ_TPP_ = 778 nm and λ_SPP_ = 798 nm in ethanol, for TPP and SPP, respectively). These optical dispersion features were related to the presence of a surface lattice array on the gold layer. In fact, the introduction of the surface lattice grating array generates a new hybrid plasmonic mode where the propagated surface plasmons from the ambient side are coupled with Bragg reflections on the nano-bumps and simultaneously coupled with Tamm plasmons from the 1D photonic crystal side. Compared to the surface lattice resonances excited from the grating side, the internal reflection configuration on the surface lattice array works as hot spots, leading to an increased electric field intensity at the interface [28]. This periodic surface nanostructure generates a new type of hybrid plasmonic excitation related to propagated surface plasmons, localized plasmons, and Bragg reflections from the surface grating array-propagated surface lattice plasmonic resonance (PSLPR). 

**Figure 4:**
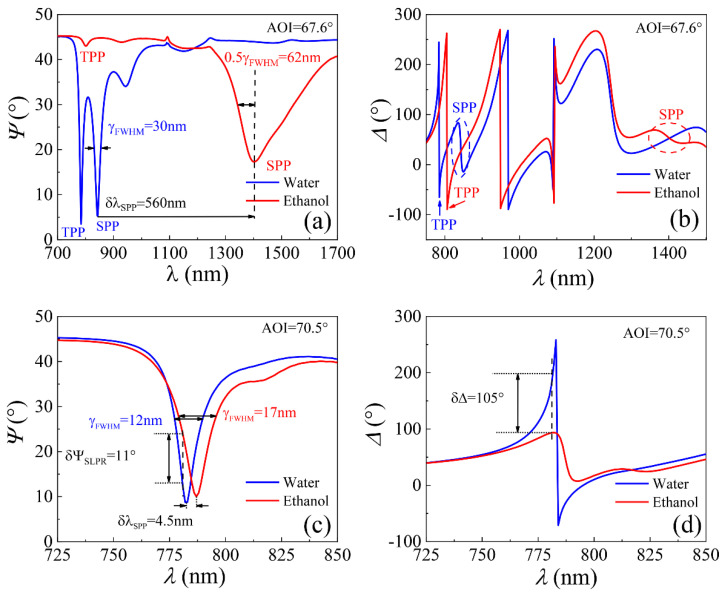
Spectra of ellipsometric parameters Ψ(λ) (**a**,**c**) and Δ(λ) (**b**,**d**) in both ambient, where the blue curve corresponds to water and the red curve to ethanol. The ellipsometric spectra for structure with thin gold layer (**a**,**b**), and for structure with gold nano-bumps (**c**,**d**). The dashed areas (blue and red) in figure (**b**) show the ellipsometric parameter Δ at the SPP position and the wavelength shift due to refractive index changes in different ambient.

The same ellipsometric measurements for the uniform gold layer were performed on the area with gold lattice. The TPP and HSLPP components of the hybrid plasmonic excitation were at fixed AOI (θ = 70.5°), and the dispersion relation lies in λ_TPP_ = 667 nm and λ_HSLPP_ = 783 nm for the deionized water and was λ_TPP_ = 683 nm and λ_HSLPP_ = 787 nm for the ethanol at the same AOI. The shifts were δλ_TPP_ = 15.5 nm and δλ_HSLPP_ = 4.5 nm in both ambient (Figure 4c,d). For the TPP resonance, such spectral shift corresponds for refractive index change δn_(λ=667nm)_ = 1.3512 − 1.331 = 0.0202 and for the HSLPP resonance shift corresponds for δn_(λ=783)_ = 1.3494 − 1.3286 = 0.0208 refractive index change. Thus, this leads to the corresponding spectral sensitivity of these resonances to refractive index unit *δλ*_TPP_/*δn* = 15.5/0.0202 = 767 nm/RIU and *δλ*_HSLPP_/*δn* = 4.5/0.0208 = 215 nm/RIU. 

It should be noted that a much wider full width at half maximum (FWHM) was registered for the SPP excitation in the hybrid mode with a uniform gold layer: in the deionized water δλ_FWHM(SPP)_ = 29.6 nm and in ethanol δλ_FWHM(SPP)_ = 124.5 nm. As a result, the Q-factor in the experimental hybrid (film) structure were Q_SPP_ = 28.4 in deionized water and Q_SPP_ = 11.3 in ethanol (Figure 4c,d). For the hybrid mode with nano-bumps array FWHM was registered: δλ_FWHM(HSLPP)_ = 63.6 nm in the deionized water and δλ_FWHM(HSLPP)_ = 46.3 nm in ethanol. The Q-factor in the experimental hybrid mode for the lattice SLPR: Q_HSLPP_ = 63.6 deionized water and Q_HSLPP_ = 46.3 in ethanol. As can be seen, the Q-factor was better for the hybrid structure with a lattice of gold nano-bumps than on the uniform gold layer. It can be explained by the lower losses of such lattice mode in which Bragg reflection compensates the phase changes of SPP on the lattice. The presence of nano-bumps lattice on the gold surface significantly decreased optical signal sensitivity to the changes of the refractive index of the ambient compared with the uniform gold layer. However, at the same time, the Q-factor increased for the sample area with lattice, which indicates decreasing losses in the metal layer for such plasmonic modes. It should be noted that high Q-factors (~150) were achieved with surface lattice plasmonic resonances (SLR) generated on ordered gold nanoparticles also with prism coupler in the deep red spectral range [20]. Even higher Q-factors for SLR were demonstrated on indium tin oxide in the infrared region (λ~5µm) [39]. The better Q-factor for plasmonic resonances with gold nano-bumps lattice increases the sensitivity of ellipsometric parameters Ψ and Δ to the refractive index changes. The ellipsometric parameter Ψ changes about ~11° meanwhile Δ changes ~105° for δn = 0.0205, which gives sensitivity S_Ψ_ ≈ 537°/RIU and S_Δ_ ≈ 5122°/RIU.

## 4. Conclusions

Summarizing the TIRE method in the Krescthmann configuration was used for the excitation of strong coupling between TPP and SPP in nanophotonic structures with 1D PC and gold layer on the top. The sensitivity properties of the uniform gold layer and modified area forming a lattice of gold nano-bumps by direct laser writing were compared. Recent studies have shown very high hybrid plasmonic mode sensitivity S_HSPP_ ≈ 26,000 nm/RIU to the refractive index on the uniform gold layer; meanwhile, the introduction of gold lattice decreases the spectral signal sensitivity but increases the Q-factor of the plasmonic resonances and also induces the generation of additional Bragg mode related to lattice period, which is not involved in the strong coupling of the hybrid TPP-SPP polaritonic mode. Despite this, the sensitivity to the ellipsometric parameters Ψ and Δ was rather high due to the increased Q-factor of the resonances. The comparison of plasmonic resonance sensitivity to the refractive index changes of hybrid TPP-SPP mode on the uniform gold layer and traditional surface plasmon resonance (SPR) have shown that hybrid plasmonic mode, due to the strong coupling effect, overcomes the SPR by about 27%. The involvement of the strong coupling effect in optical sensing development of plasmonic-based sensors opens new possibilities to the advanced detection of proteins interaction, for instance, a higher sensitivity of the signal, tuning of chemical reaction rates, more than one plasmonic mode monitoring in real-time, and others.

## Data Availability

Not applicable.

## References

[1] Barnes W.L. (2006). Surface Plasmon–Polariton Length Scales: A Route to Sub-Wavelength Optics. J. Opt. A Pure Appl. Opt..

[2] Liedberg B., Nylander C., Lundström I. (1995). Biosensing with Surface Plasmon Resonance—How It All Started. Biosens. Bioelectron..

[3] Kravets V.G., Schedin F., Jalil R., Britnell L., Gorbachev R.V., Ansell D., Thackray B., Novoselov K.S., Geim A.K., Kabashin A.V. (2013). Singular Phase Nano-Optics in Plasmonic Metamaterials for Label-Free Single-Molecule Detection. Nat. Mater..

[4] Tsurimaki Y., Tong J.K., Boriskin V.N., Semenov A., Ayzatsky M.I., Machekhin Y.P., Chen G., Boriskina S.V. (2018). Topological Engineering of Interfacial Optical Tamm States for Highly Sensitive Near-Singular-Phase Optical Detection. ACS Photonics.

[5] Nishijima Y., To N., Balčytis A., Juodkazis S. (2022). Absorption and Scattering in Perfect Thermal Radiation Absorber-Emitter Metasurfaces. Opt. Express.

[6] Koch U., Uhl C., Hettrich H., Fedoryshyn Y., Moor D., Baumann M., Hoessbacher C., Heni W., Baeuerle B., Bitachon B.I. (2021). Plasmonics—High-Speed Photonics for Co-Integration with Electronics. Jpn. J. Appl. Phys..

[7] Ma R.-M., Oulton R.F., Sorger V.J., Bartal G., Zhang X. (2011). Room-Temperature Sub-Diffraction-Limited Plasmon Laser by Total Internal Reflection. Nat. Mater..

[8] Kreibig U., Vollmer M. (1995). Optical Properties of Metal Clusters.

[9] Homola J., Yee S.S., Gauglitz G. (1999). Surface Plasmon Resonance Sensors: Review. Sens. Actuators B Chem..

[10] Kravets V.G., Kabashin A.V., Barnes W.L., Grigorenko A.N. (2018). Plasmonic Surface Lattice Resonances: A Review of Properties and Applications. Chem. Rev..

[11] Reshetnyak V., Pinkevych I., Bunning T., Evans D. (2021). Influence of Rugate Filters on the Spectral Manifestation of Tamm Plasmon Polaritons. Materials.

[12] Plikusienė I., Bužavaitė-Vertelienė E., Mačiulis V., Valavičius A., Ramanavičienė A., Balevičius Z. (2021). Application of Tamm Plasmon Polaritons and Cavity Modes for Biosensing in the Combined Spectroscopic Ellipsometry and Quartz Crystal Microbalance Method. Biosensors.

[13] Gupta N.K., Tiwari A.K., Wanare H., Ramakrishna S.A. (2021). Near Singular-Phase Optical Biosensing with Strongly Coupled Modes of a Plasmonic–Photonic Trimer. J. Opt..

[14] Sasin M.E., Seisyan R.P., Kalitteevski M.A., Brand S., Abram R.A., Chamberlain J.M., Egorov A.Y., Vasil’ev A.P., Mikhrin V.S., Kavokin A.V. (2008). Tamm Plasmon Polaritons: Slow and Spatially Compact Light. Appl. Phys. Lett..

[15] Balevicius Z., Baskys A. (2019). Optical Dispersions of Bloch Surface Waves and Surface Plasmon Polaritons: Towards Advanced Biosensors. Materials.

[16] Sinibaldi A., Danz N., Descrovi E., Munzert P., Schulz U., Sonntag F., Dominici L., Michelotti F. (2012). Direct Comparison of the Performance of Bloch Surface Wave and Surface Plasmon Polariton Sensors. Sens. Actuators B Chem..

[17] Bužavaitė-Vertelienė E., Maciulis V., Anulytė J., Tolenis T., Baskys A., Plikusiene I., Balevičius Z. (2022). Total Internal Reflection Ellipsometry Approach for Bloch Surface Waves Biosensing Applications. Biosensors.

[18] Amra C., Zerrad M., Lemarchand F., Lereu A., Passian A., Zapien J.A., Lequime M. (2018). Energy Density Engineering via Zero-Admittance Domains in All-Dielectric Stratified Materials. Phys. Rev. A.

[19] Niu D., Zerrad M., Lereu A., Moreau A., Lumeau J., Zapien J.A., Passian A., Aubry V., Amra C. (2020). Excitation of Bloch Surface Waves in Zero-Admittance Multilayers for High-Sensitivity Sensor Applications. Phys. Rev. Appl..

[20] Kravets V.G., Schedin F., Kabashin A.V., Grigorenko A.N. (2010). Sensitivity of Collective Plasmon Modes of Gold Nanoresonators to Local Environment. Opt. Lett..

[21] Anulytė J., Bužavaitė-Vertelienė E., Vertelis V., Stankevičius E., Vilkevičius K., Balevičius Z. (2022). Influence of a Gold Nano-Bumps Surface Lattice Array on the Propagation Length of Strongly Coupled Tamm and Surface Plasmon Polaritons. J. Mater. Chem. C.

[22] Bužavaitė-Vertelienė E., Vertelis V., Balevičius Z. (2021). The Experimental Evidence of a Strong Coupling Regime in the Hybrid Tamm Plasmon-Surface Plasmon Polariton Mode. Nanophotonics.

[23] Afinogenov B.I., Bessonov V.O., Nikulin A.A., Fedyanin A.A. (2013). Observation of Hybrid State of Tamm and Surface Plasmon-Polaritons in One-Dimensional Photonic Crystals. Appl. Phys. Lett..

[24] Törmä P., Barnes W.L. (2015). Strong Coupling between Surface Plasmon Polaritons and Emitters: A Review. Rep. Prog. Phys..

[25] Pelton M., Storm S.D., Leng H. (2019). Strong Coupling of Emitters to Single Plasmonic Nanoparticles: Exciton-Induced Transparency and Rabi Splitting. Nanoscale.

[26] Kravets V.G., Schedin F., Grigorenko A.N. (2008). Extremely Narrow Plasmon Resonances Based on Diffraction Coupling of Localized Plasmons in Arrays of Metallic Nanoparticles. Phys. Rev. Lett..

[27] Stankevičius E., Vilkevičius K., Gedvilas M., Bužavaitė-Vertelienė E., Selskis A., Balevičius Z. (2021). Direct Laser Writing for the Formation of Large-Scale Gold Microbumps Arrays Generating Hybrid Lattice Plasmon Polaritons in Vis–NIR Range. Adv. Opt. Mater..

[28] Sarkar M., Besbes M., Moreau J., Bryche J.-F., Olivéro A., Barbillon G., Coutrot A.-L., Bartenlian B., Canva M. (2015). Hybrid Plasmonic Mode by Resonant Coupling of Localized Plasmons to Propagating Plasmons in a Kretschmann Configuration. ACS Photonics.

[29] Hoang C.V., Hayashi K., Ito Y., Gorai N., Allison G., Shi X., Sun Q., Cheng Z., Ueno K., Goda K. (2017). Interplay of Hot Electrons from Localized and Propagating Plasmons. Nat. Commun..

[30] Buzavaite-Verteliene E., Plikusiene I., Tolenis T., Valavicius A., Anulyte J., Ramanavicius A., Balevicius Z. (2020). Hybrid Tamm-Surface Plasmon Polariton Mode for Highly Sensitive Detection of Protein Interactions. Opt. Express.

[31] Balevičius Z. (2020). Strong Coupling between Tamm and Surface Plasmons for Advanced Optical Bio-Sensing. Coatings.

[32] Arwin H., Poksinski M., Johansen K. (2004). Total Internal Reflection Ellipsometry: Principles and Applications. Appl. Opt..

[33] Arwin H., Hinrichs K., Eichhorn K.-J. (2014). TIRE and SPR-Enhanced SE for Adsorption Processes. Ellipsometry of Functional Organic Surfaces and Films.

[34] Balevicius Z., Makaraviciute A., Babonas G.-J., Tumenas S., Bukauskas V., Ramanaviciene A., Ramanavicius A. (2013). Study of Optical Anisotropy in Thin Molecular Layers by Total Internal Reflection Ellipsometry. Sens. Actuators B Chem..

[35] Balevicius Z., Baleviciute I., Tumenas S., Tamosaitis L., Stirke A., Makaraviciute A., Ramanaviciene A., Ramanavicius A. (2014). In Situ Study of Ligand–Receptor Interaction by Total Internal Reflection Ellipsometry. Thin Solid Films.

[36] Moening J.P., Thanawala S.S., Georgiev D.G. (2009). Formation of High-Aspect-Ratio Protrusions on Gold Films by Localized Pulsed Laser Irradiation. Appl. Phys. A.

[37] Itapu S., Borra V., Georgiev D.G. (2020). Laser-Based Fabrication of Microstructures on Nickel Thin Films and Its Applications in On-Chip Thin Film Inductors. IEEE Trans. Nanotechnol..

[38] J. A. Woollam Company (2020). CompleteEase.

[39] Rhodes C., Franzen S., Maria J.-P., Losego M., Leonard D.N., Laughlin B., Duscher G., Weibel S. (2006). Surface Plasmon Resonance in Conducting Metal Oxides. J. Appl. Phys..

